# Effects of Sintering Temperature on Crystallization and Fabrication of Porous Bioactive Glass Scaffolds for Bone Regeneration

**DOI:** 10.1038/s41598-017-06337-2

**Published:** 2017-07-20

**Authors:** E. P. Erasmus, O. T. Johnson, I. Sigalas, J. Massera

**Affiliations:** 1 African Material Science and Engineering Network A Carnegie-IAS RISE Network, University of the Witwatersrand, Johannesburg, South Africa; 2 University of the Witwatersrand, School of Chemical and Metallurgical Engineering, Johannesburg, South Africa; 3 DST/NRF Centre of Excellence in Strong Materials, University of the Witwatersrand, Johannesburg, South Africa; 4 University of Namibia, Department of Mining and Metallurgical Engineering, Ongwediva, Namibia; 5 Tampere University of Technology, BioMediTech and Faculty of Biomedical Sciences and Engineering, Tampere, Finland

## Abstract

In this work the sintering ability of borosilicate (S53B50), borophosphate (P40B10) and phosphate (Sr) bioactive glasses was investigated. The glass powders were crushed and sintered in air at a heating rate of 10 °C/min for 2 hours at sintering temperatures between 480 °C–600 °C. The aim was to define the optimum sintering temperature prior to glass crystallization. The density of the samples was found to decrease when the temperature was increased up to 580 °C; probably due to the inhibition of the viscous flow of the particles during sintering thereby reducing the densification of the material. Such low porosity is not suitable in tissue engineering. To process highly porous scaffolds with porosity required for scaffold applicable to tissue engineering, the powders were further mixed with 60 vol.% and 70 vol.% of NH_4_(HCO_3_) foaming agent. Meanwhile, the density of the samples sintered with NH_4_(HCO_3_) was found to decrease with an increase in NH_4_(HCO_3_) content. This indicates an increase in porosity of the samples. The glass compositions reached an open porosity of more than 60% at the addition of 70 vol.% NH_4_(HCO_3_). In addition, SEM micrograph revealed large pores with good interconnection between the pores.

## Introduction

The large number of bone defects caused from a disease or trauma still remains a significant clinical challenge. At present, autografts and allografts are mainly used in the repair of defected bones. These grafts offered all the desired properties required for bone repair and regeneration, which are osteoconductivity, osteogenesis, and osteoinductivity. However, bone grafts are associated with high operating costs for harvesting the graft, limited availability, donor site morbidity and complications including infection, pain, and hematoma^1, 2^. On the other hand, allografts are subject to cleaning and preparation processes designed to remove cells to minimize immune response. In addition, these process techniques potentially reduce the osteoinductivity, osteoconductivity, and mechanical strength of the graft^3^. Therefore investigation of biomaterials which could be used in the repair and regeneration of bones remains of paramount importance.

One such biomaterial is bioglass^®^ (45S5) with composition in the SiO_2_-CaO-Na_2_O-P_2_O_5_ system discovered by Hench *et al*.^4^ Bioglass^®^, as well as other similar glass compositions, such as BonAlive(R) (S53P4) were found to rapidly form a chemical bond with bone through the precipitation of a hydroxyapatite layer at the glass surface. The mechanism enabling strong bone/glass interface has been reviewed in detail in the past^5^. However, typical silicate bioactive glasses have two major drawbacks despite their bond forming abilities. These include their poor mechanical properties (caused by their amorphous nature) and their tendency to crystallize during sintering^6, 7^.

According to Filho *et al*.^8^, the presence of crystalline phases formed during heat treatment tend to retard the formation of the hydroxyapatite layer, hence glass becomes less bioactive in simulated body fluid. Therefore, crystallization should be prevented whenever possible. This becomes an issue to overcome, as in order to obtain clinically relevant construct, the graft should be a 3D structure with large pore size (>100 µm) and porosity (>70%). The construct should have mechanical properties similar to the tissue to be replaced and should be prepared in a reproducible manner. Typical 3D scaffold manufacturing involves a firing and sintering step which will lead, in most cases, to crystallization of the glass^9^.

Over the years new glass compositions with no or lower SiO_2_ content have been developed. Phosphate and borophosphate glasses have been found to not only be promising as biomaterials^10, 11^, but also allows hot forming at temperature below their crystallization domain^12, 13^. Borosilicate glasses also demonstrated to have thermal properties enabling hot forming more readily than typical silicate bioactive glasses^14^. Not only these glasses have enhanced hot forming domain but they were also found to promote cell adhesion and proliferation as well as differentiation into bone cells^15^.

Locs *et al*.^16^, studied the preparation of porous ceramics by viscous slurry foaming using hydroxyapatite powder and ammonium hydrogen carbonate as the foaming agent for the generation of pores in the glycerol-based viscous ceramic slurry. Open and total porosity were found to increase from 25 to 69% and from 32 to 73% when NH_4_(HCO_3_) content was varied from 0 to 2.75 wt.%. The sintered ceramics were found to have well-connected, irregularly shaped pores. Meanwhile, Kazutaka *et al*.^17^, produced highly porous alumina based ceramics by incorporating polymethylmethacrylate (PMMA) microspheres with different diameters as a template and MgO or SiC powder as a sintering aid and subsequent calcinations at 1600 °C. The sintered ceramics formed spherical pores which correlated to the morphology of the PMMA microspheres. Highly porous and mechanically strong alumina-based ceramics having an open porosity of 62%, a connected space size of 1.3 µm, and a compressive strength of 147.6 MPa were fabricated by incorporating PMMA microspheres with a mean particle size of 22.6 µm and an appropriate amount of SiC

This work aims to determine the time-temperature conditions that allow for the sintering of a borosilicate (S53B50), borophosphate (P40B10) and phosphate (Sr) with minor or no crystallization as well as the fabrication of porous scaffolds using NH_4_(HCO_3_).

## Results

### Thermal properties

The three glasses under investigation were processed with the composition presented in Table 1.

**Table 1:** Nominal composition of S53B50, P40B10 and Sr glasses presented in molar percent (mol.%).

Composition (mol.%)	S53B50	P40B10	Sr
SiO_2_	26.93	0	0
Na_2_O	22.66	10.00	10.00
CaO	21.77	20.00	0
B_2_O_3_	26.93	10.00	0
P_2_O_5_	1.72	40.00	50.00
SrO	0	20.00	40.00

**Figure 1:**
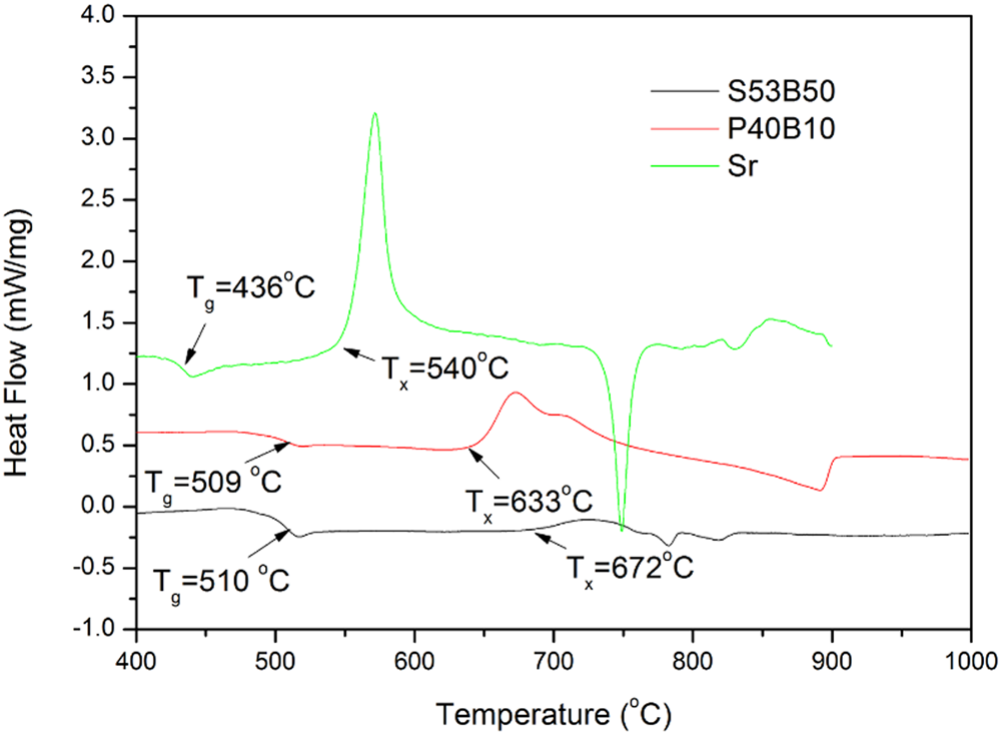
DTA thermograph of the glasses of investigation.

Figure 1 shows the DTA trace of the glasses S53B50, P40B10 and Sr recorded at a heating rate of 10 °C/min on a powder sample of 125–250 µm particle size. The glass transition (T_g_) is taken at the inflection point of the endotherm (obtained by taking the first derivative of the curve) and T_x_ is defined as the onset of the crystallization peak. The working range (or hot forming domain) was defined as ΔT=T_x_﻿−T_g_. ﻿In this study, the crystallization temperature (T_p_) which is at the maximum of the exothermic peak was not measured. The quantified values are presented in Table 2.

**Table 2:** Glass transition temperature (T_g_), onset of crystallization temperature (T_x_), and working range (∆T) of S53B50, P40B10 and Sr bioactive glasses, as determined by differential thermal analysis (DTA).

Characteristic temperature (°C)	S53B50	P40B10	Sr
Glass transition temperature (T_g_ ± 2 °C)	510	509	436
Onset of crystallization temperature (T_x_ ± 2 °C)	672	633	540
Working range (∆T = T_x_ – T_g_ ± 4 °C)	162	124	104

### Powder morphology of bioactive glasses

**Figure 2:**
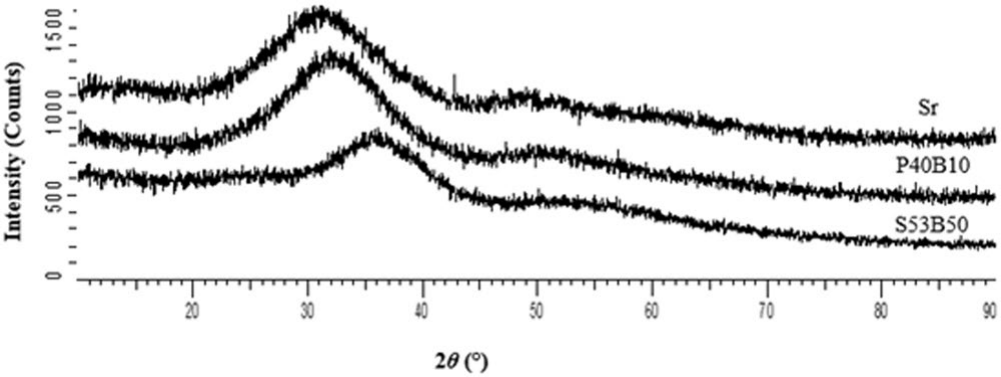
X-ray diffraction patterns of S53B50, P40B10 and Sr bioactive glasses after milling.

Figure 2 shows the XRD patterns of the as prepared powders prepared without NH_4_(HCO_3_). The patterns showed that the bioactive glasses were amorphous and free of any significant crystalline phases.

**Figure 3:**
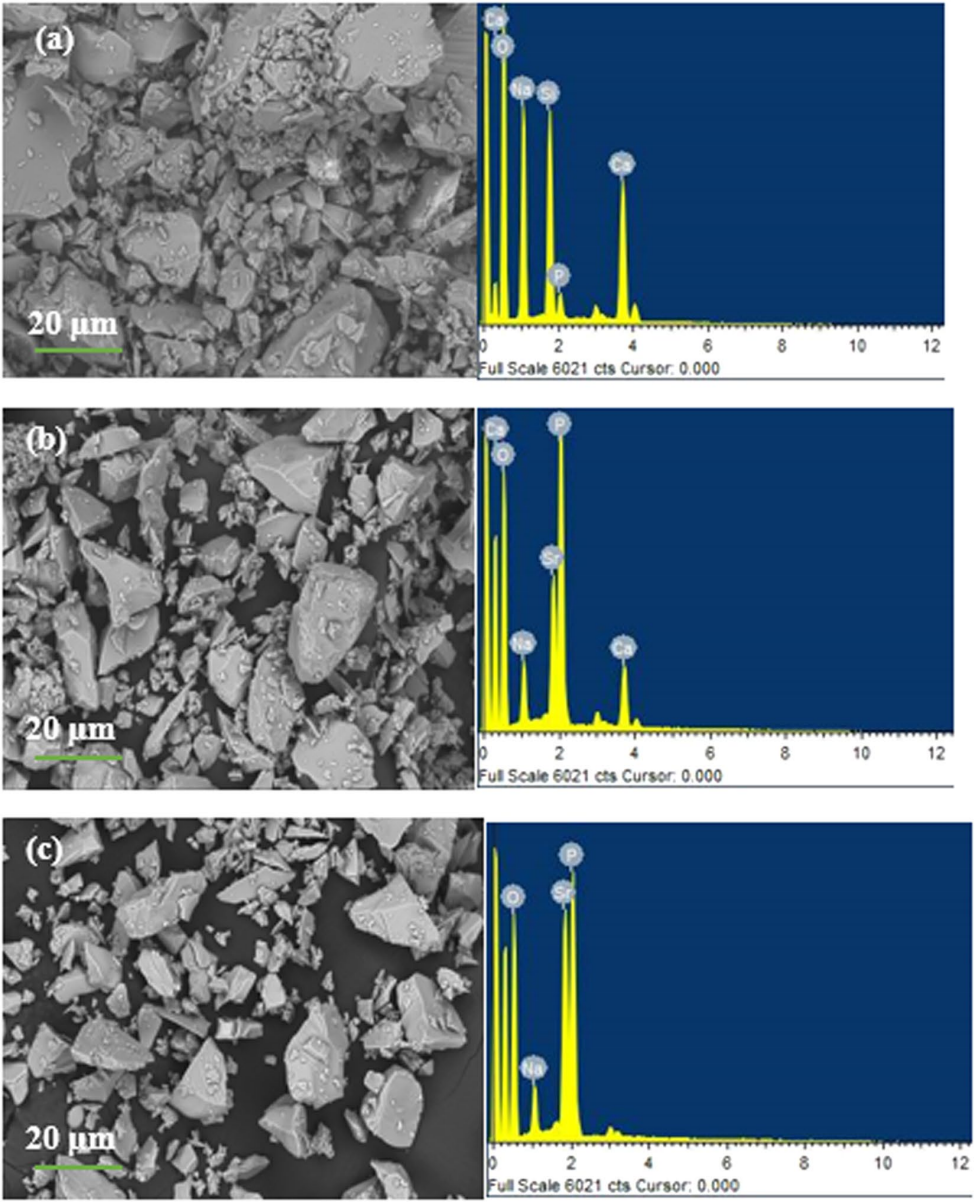
SEM images of (**a**) S53B50 (**b**) P40B10 and (**c**) Sr bioactive glass powder showing the particle size, shape and EDS analysis.

Figure 3 presents the SEM micrographs of the powders. It reveals that the bioactive glass powders were composed of particles exhibiting irregular shapes and a wide size distribution, with small particles tending to attach themselves to the surfaces of the larger ones. The average particle size obtained after milling for glasses S53B50, P40B10 and Sr were 20.54 µm, 18.37 µm and 26.37 µm respectively. EDS analysis was performed and the glass composition was found to be in agreement with the nominal one (Table 1).

Similar results were observed for the bioactive glass powders prepared with NH_4_(HCO_3_), it is important to note that the NH_4_HCO_3_ was added after milling. The glasses were amorphous and had no significant crystalline phases after milling and the glass particles also exhibited irregular shapes with a wide size distribution (results not shown). The average particle size of S53B50, P40B10 and Sr were 39.9 µm, 32.8 µm and 46.3 µm respectively, with this said, it is noteworthy to mention that the large particle size obtained for these glasses was due to the small force that was used during crushing compared to the force applied during crushing of the glasses that were prepared without NH_4_(HCO_3_). This increase in particle size was crucial in that, fine particles were reported to enhance crystallization of bioactive glass powders^7^.

### Densification and Microstructural analysis of samples sintered with/without NH_4_(HCO_3_)

**Figure 4:**
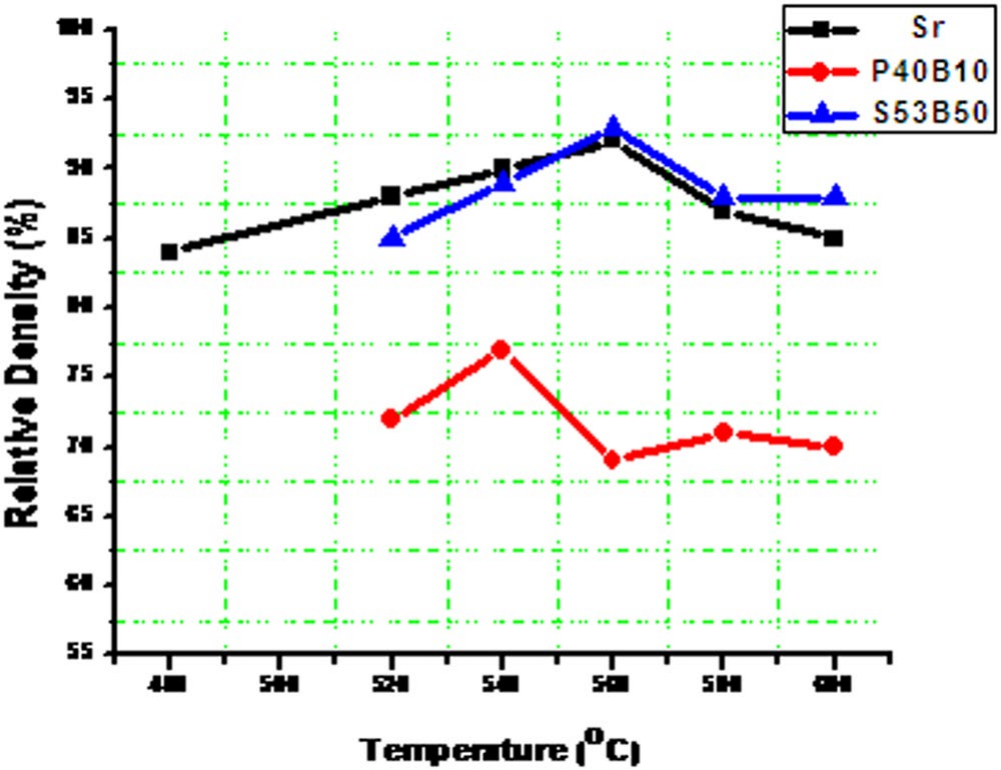
Relative density of S53B50, P40B10 and Sr bioactive glasses as a function of temperature.

Samples were sintered at temperatures between 480 and 600 °C. The obtained densities of the sintered bioactive glasses without NH_4_(HCO_3_) are shown in Fig. 4. The density of the sintered samples for glass S53B50 and Sr was found to increase up to 560 °C and decreased as the sintering temperature was further increased to 600 °C. The decrease in density may be attributed to the inhibition of the viscous flow of the particles caused by surface nucleation and/or crystallization; hence, particles may not fuse together properly during sintering resulting in poor densification of the material.

Glass P40B10 samples showed the same trend, but with slightly lower densification than S53B50 and Sr, this may be due to high activation energy for viscous flow for this particular glass composition. The maximum densification (theoretical density-TD %) achieved for glasses S53B50, P40B10 and Sr were 93%, 72% and 92% respectively.

**Figure 5:**
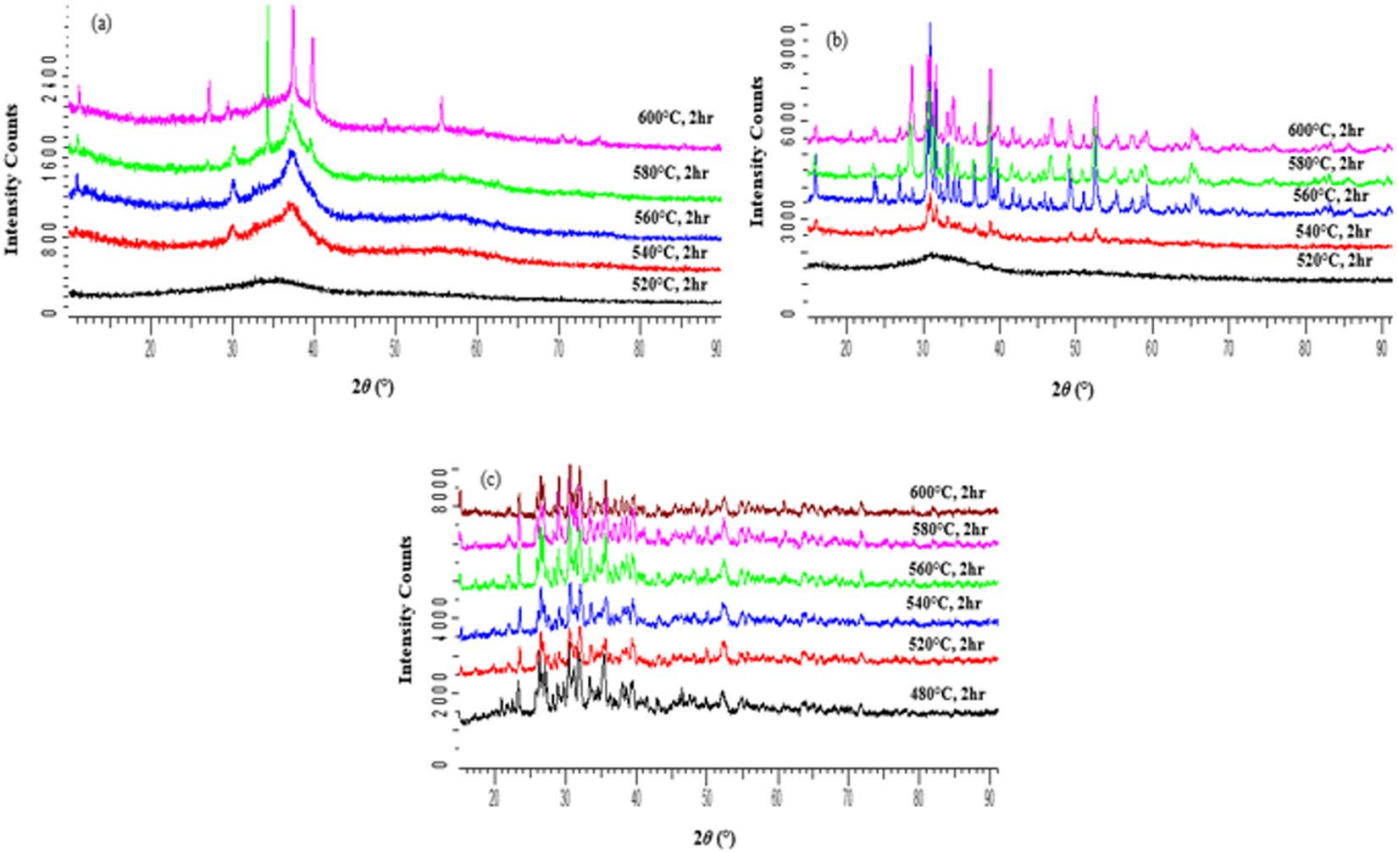
X-ray diffraction patterns of glass (**a**) S53B50 (**b**) P40B10 and (**c**) Sr as a function of temperature.

Figure 5 shows the XRD patterns of the bioactive glasses sintered without NH_4_(HCO_3_) for each sintering temperature. As the sintering temperature was increased, the samples began to crystallize as evidenced by the presence of sharp diffraction peaks. As expected, the crystallization occurs at lower temperature than T_x_ due to the small particle size used compared to the one analyzed by DTA.

**Figure 6:**
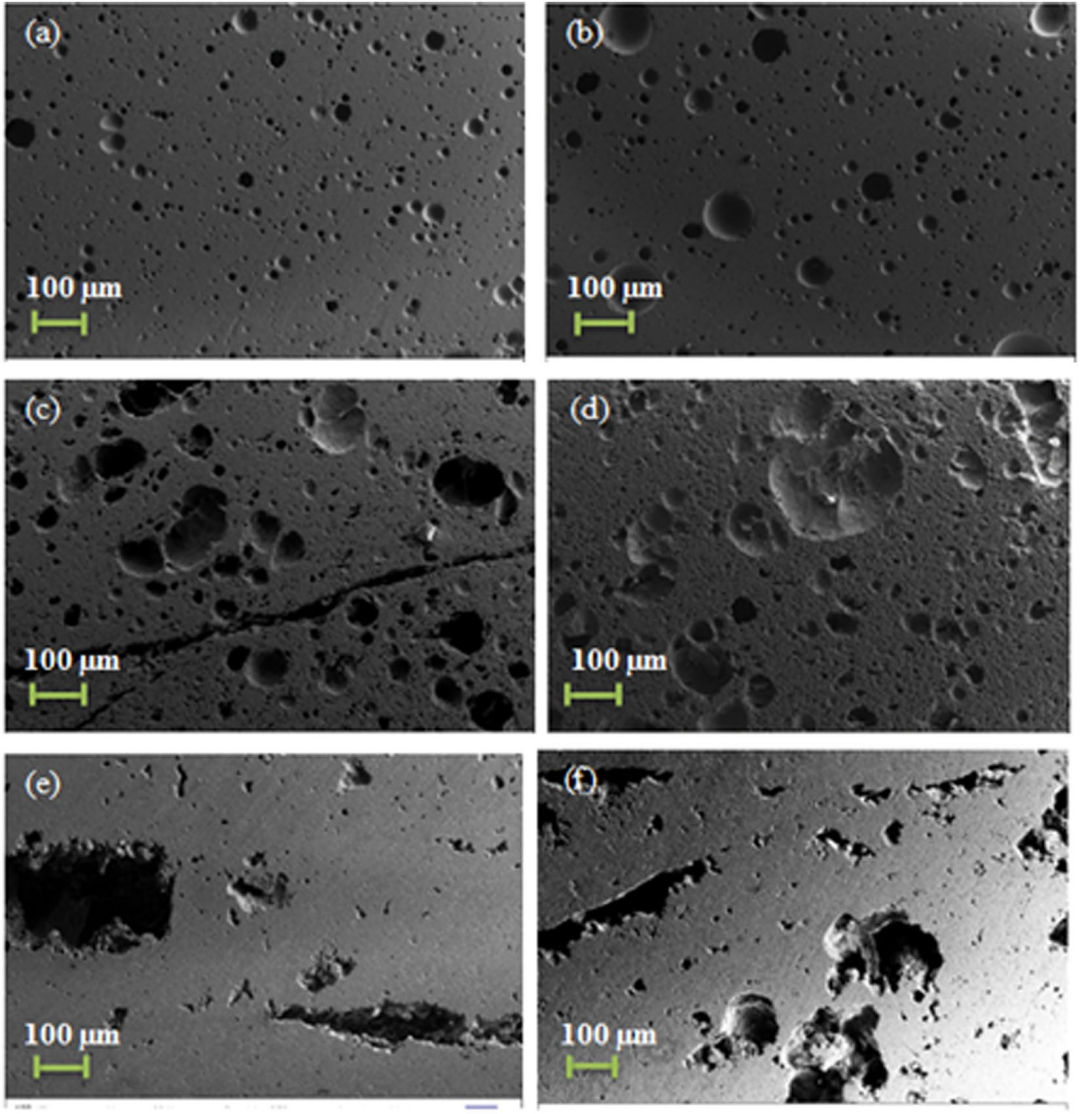
SEM micrographs of the cross section of samples sintered at 580 °C, 600 °C for glasses S53B50 (**a**,**b**), P40B10 (**c**,**d**) and Sr (**e**,**f**).

**Figure 7:**
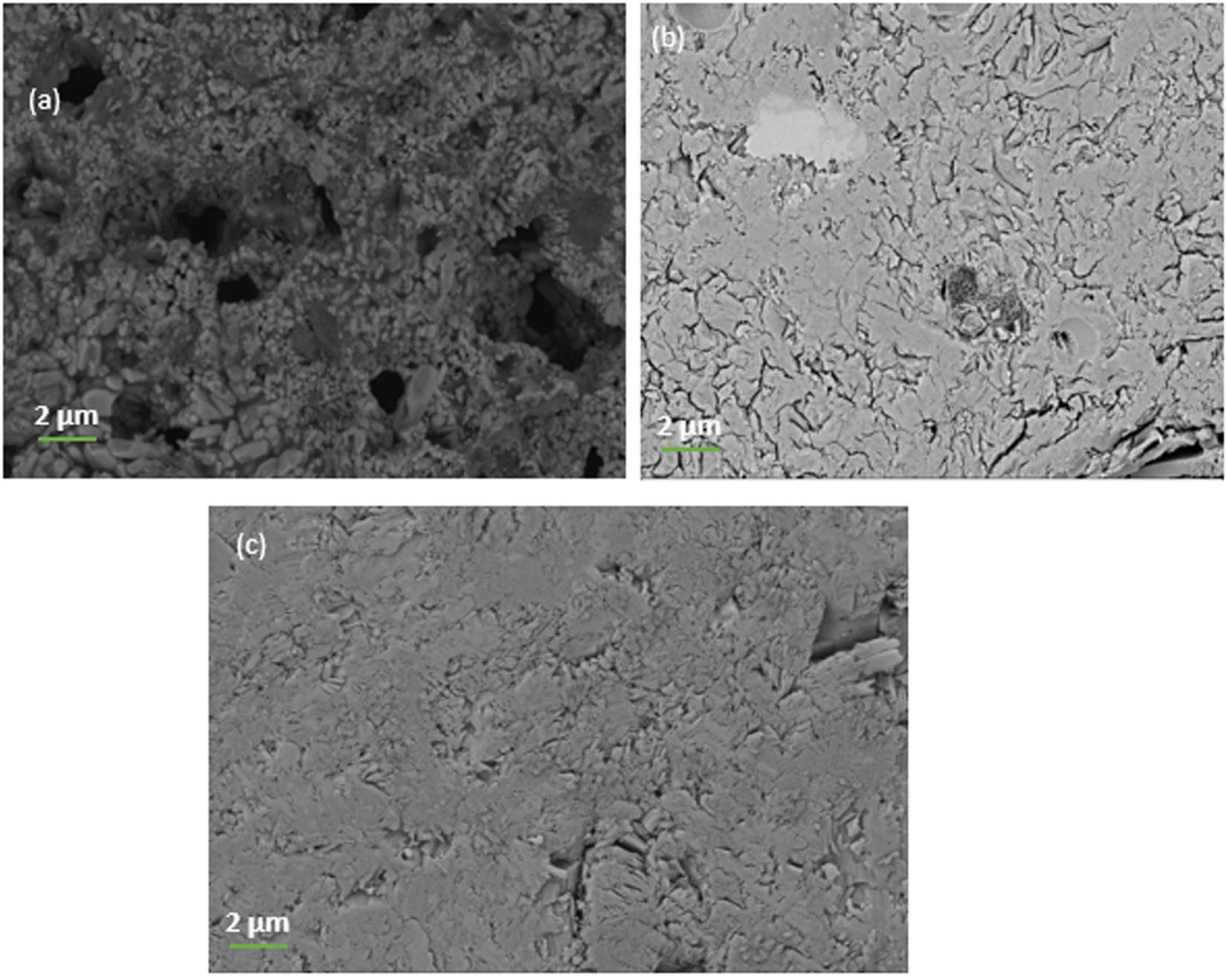
SEM micrographs of the cross section of glass samples sintered at 600 °C for glass (**a**) P40B10 (**b**) S53B50 and (**c**) Sr.

SEM investigations of the sintered bodies’ cross sections in Fig. 6 reveal dense microstructures which correlates with the densification results in Fig. 4. However, the presence of micropores is also evident which could have contributed to the reduction in the density of the samples. These micropores might be residual porosity as a result of the incomplete sintering process. In addition, Fig. 7 shows the presence of crystals formed on the glass surface during sintering as a result of crystallization. As mentioned earlier, this action should be controlled/minimized in order to retain the bioactivity of the glasses.

The porosity from the sintered samples without NH_4_(HCO_3_) was too low for tissue engineering application therefore; a second batch of experiments was conducted with the addition of NH_4_(HCO_3_) with the aim of increasing the porosity of the samples. Table 3 summarizes the density and porosity of the obtained scaffolds.

**Table 3:** Composition, Density and Open porosity of sintered scaffolds.

Composition	Temperature (°C), Time (Hr)	Density (g/cm^3^)	Theoretical density (%) TD	Porosity (%)
S53B50 – 60 vol.% NH_4_(HCO_3_)	540, 1	1.07	41	59
S53B50 – 70 vol.% NH_4_(HCO_3_)	540, 1	0.86	33	67
P40B10 – 60 vol.% NH_4_(HCO_3_)	540, 1	1.19	41	59
P40B10 – 70 vol.% NH_4_(HCO_3_)	540, 1	0.95	32	68
Sr – 60 vol.% NH_4_(HCO_3_)	500, 1	1.02	36	64
Sr – 70 vol.% NH_4_(HCO_3_)	500, 1	0.79	28	72

**Figure 8:**
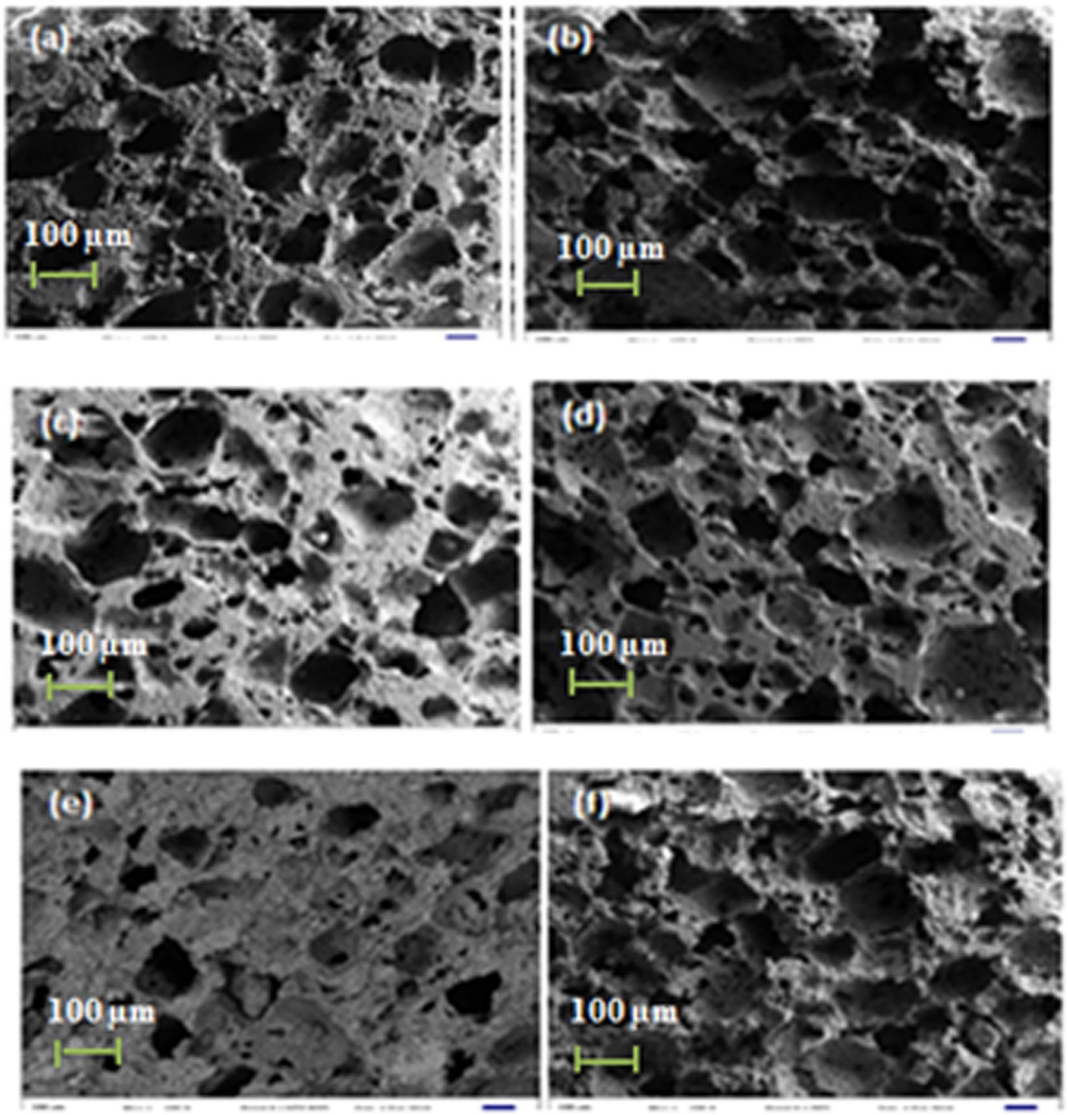
SEM micrographs of porous S53B50, P40B10 and Sr bioactive scaffolds mixed with (**a**), (**c**) and (**e**) 60 vol.% (NH_4_)HCO_3_ and (**b**,**d** and **f**) 70 vol.% (NH_4_)HCO_3_ respectively, sintered at 540 °C and 500 °C for 1 hour respectively.

The porosity of the scaffolds was found to increase as the NH_4_(HCO_3_) content was increased. A similar trend was observed by Locs *et al*.^16^, whereby the open and total porosity were found to increase from 25 to 69% and from 32 to 73% when NH_4_(HCO_3_) content was varied from 0 to 2.75 wt.%. The glass compositions reached an open porosity of more than 60% at the addition of 70 vol.% NH_4_(HCO_3_), with glass Sr obtaining the highest open porosity of 72% TD. SEM analysis of the cross sections in Fig. 8 reveal a porous microstructure with well-connected open porosity and irregularly shaped pores; hence, the low density. The formation of such porosity is caused by the high volume expansion (more than 1000 times) ratio during decomposition of NH_4_(HCO_3_)^16^. It is known that porosity decreases the density of the materials as confirmed by the densification results. The interconnection between the pores is clearly presented in the SEM micrographs. Homogeneity of pore distribution is also evident on the cross section of the scaffolds. All the glasses had a similar microstructure.

In addition, the pore walls were composed of dual microstructures (results not shown) with micro pores observed on the pore struts as well as all over the surface; this was probably due to the gas released during the sintering process. However, the micro pores were only clearly visible on glass P40B10 and Sr. The presence of micro pores is an important feature of these scaffolds, as it is known that small pores favour cell adhesion and allow the physiological fluids to enter the inner part of the scaffold^18^.

**Figure 9:**
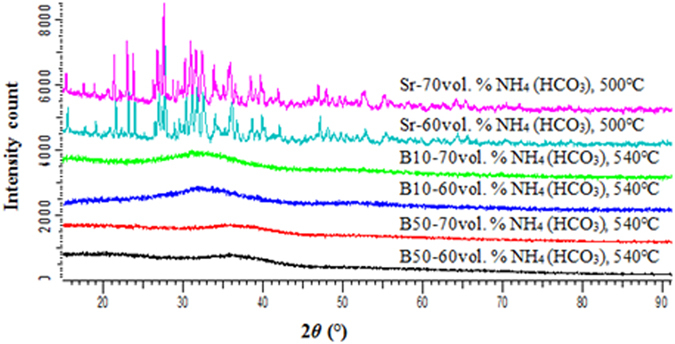
XRD patterns of the porous glass scaffolds for S53B50, P40B10 and Sr.

The scaffolds were investigated by XRD to check for the presence of any crystalline phase formed during sintering. The XRD patterns for glass S53B50 and P40B10; shown in Fig. 9 had a broad shallow peak which indicates the glasses retained the amorphous state during sintering, while glass Sr still showed a clear sign of crystallization. However it is worth mentioning that the intensity of the diffraction pattern was more pronounced when the glass was sintered without foaming agent.

The particle size is known to play a critical role in sintering as well as in crystallization^19^. Particle sizes for samples prepared without NH_4_(HCO_3_) were in average 20.5 µm, 18.4 µm and 26.4 µm for glass S53B50, P40B10 and Sr respectively, while the average particle sizes for the glass powders sintered with NH_4_(HCO_3_) was 39.9 µm, 32.8 µm and 46.3 µm for S53B50, P40B10 and Sr respectively. Fine particles are known to increase the number of nucleation sites by increasing surface to volume ratio thus increasing the crystallization kinetics of glasses with surface initiated crystallization^6, 19^. Aside from the particles size which might affect the crystallization kinetics, the presence of carbon, from the binder, at the particles surface may inhibit crystallization.

**Figure 10:**
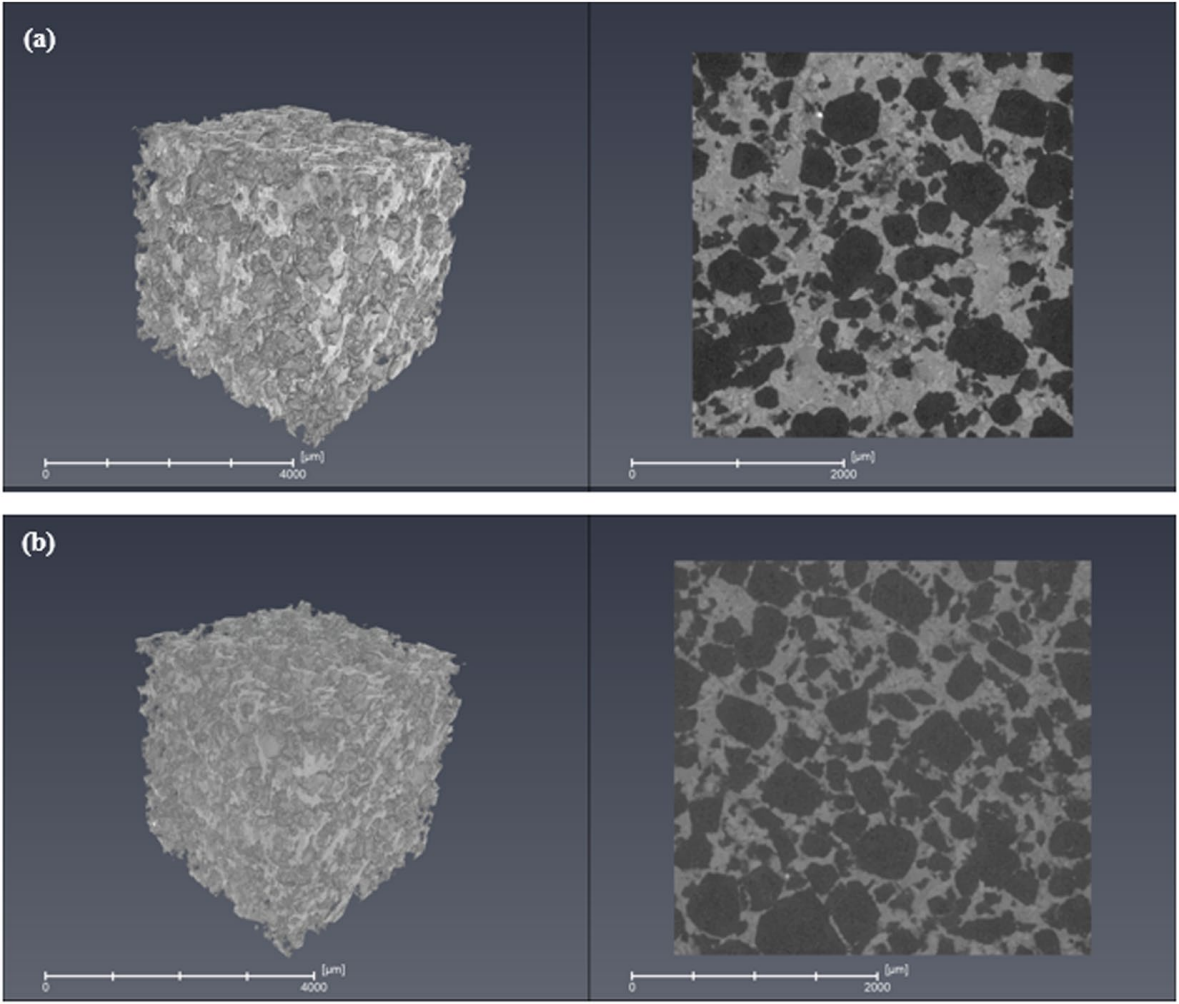
An X-ray micro tomography (µCT) image of S53B50 glass scaffold mixed with (**a**) 60 vol.% and (**b**) 70 vol.% NH_4_(HCO_3_).

For a scaffold to be ideal for any tissue engineering application, the most important architectural feature of the scaffold is the interconnecting pore apertures of the pore network. A modal (Dmode) interconnected pore size of 100 µm or more is required in order for the scaffold to allow tissue ingrowth and vascularization. The 3-D µCT images of the structure of the optimized scaffolds at different NH_4_(HCO_3_) content are shown in Fig. 10 for glass S53B50. From the images, it is evident that the pores generated and porosity; increased with the NH_4_(HCO_3_) as observed with the SEM analysis. There is also clear visual evidence of the high interconnectivity and porosity of the scaffolds. In addition, there appears to be a homogenous distribution of pores which are distributed uniformly all over the scaffolds. This trend was observed for glass P40B10 and Sr scaffolds.

**Figure 11:**
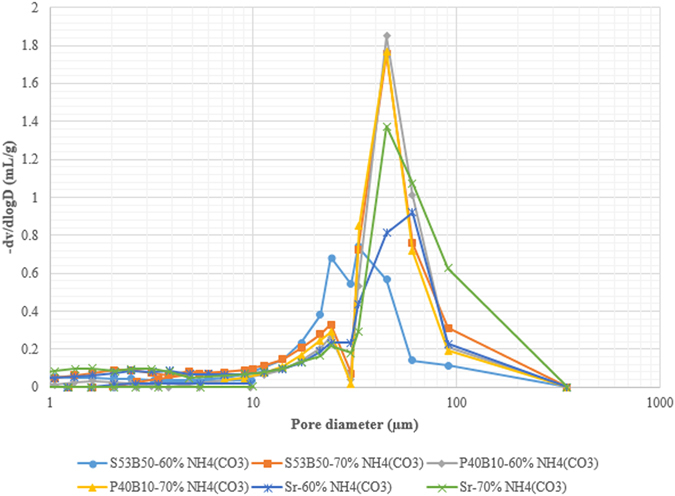
Pore size distribution of the glass scaffolds at different NH_4_(HCO_3_) content.

Figure 11 presents the pore size distribution of the scaffolds obtained from mercury intrusion porosimetry. The vertical axis is a derivative of the volume of mercury intruded into the scaffolds relative to the pore diameter^20^. The figure show that all scaffold have broad pore distribution in the range 0.003 µm – 100 µm with a D_mode_ at ~50 µm.

Table 4 summarises the characterization data of the scaffolds obtained from the analysis. From the results, as expected, the average pore size and porosity increased as a function of NH_4_(HCO_3_) content.

**Table 4:** Composition, Average pore diameter and % Porosity of scaffolds.

Composition	From porosimetry		From µCT		
	Median pore diameter (µm)	Porosity (%)	Average pore diameter (µm)	Maximum pore diameter µm	Porosity (%)
S53B50 – 60 vol.% NH_4_(HCO_3_)	33	55	257.2	513.4	54.1
S53B50 – 70 vol.% NH_4_(HCO_3_)	53	68	257.5	518.5	61.8
P40B10 – 60 vol.% NH_4_(HCO_3_)	55	66	259.8	570.0	46.9
P40B10 – 70 vol.% NH_4_(HCO_3_)	53	63	262.3	509.3	60.2
Sr – 60 vol.% NH_4_(HCO_3_)	56	67	243.8	482.0	58.0
Sr – 70 vol.% NH_4_(HCO_3_)	69	76	285.5	519.0	64.7

From the results, as expected, the average pore size and porosity increased as a function of NH_4_(HCO_3_) content. The pore size obtained from porosimetry is significantly lower than values obtained by µCT. However, one should keep in mind that the porosimetry does not provide measure of the pores but rather, the interconnected size^21, 22^. In addition it is noteworthy to mention the similarities in the porosity obtained in Tables 3 and 4 respectively.

## Discussion

All the DTA traces exhibited an endothermic event corresponding to the glass transition and an exothermic event corresponding to the crystallization of the glass. From the thermograph, T_g_ (glass transition temperature) and T_x_ (onset of crystallization) were recorded and are presented in Fig. 1. ∆T (∆T = T_x_ − T_g_), also presented in Table 2, is a gauge to glass resistance to crystallization^6^. According to Brauer^23^, crystallization of bioactive glasses can be controlled by increasing the working range which is the temperature difference between crystallization and the glass transition. This stability range, ∆T, is the temperature range in which glass viscosity allows for sintering, fiber drawing, or other processing. A large temperature gap between glass transition and onset of crystallization suggests that viscous flow is more likely to occur prior to the crystallization. A small temperature gap between glass transition and onset of crystallization suggests a nucleation close to the glass transition temperature and therefore more risk of nucleation and growth of crystals during sintering, prior or during sintering. Furthermore, it is noteworthy that all glasses are expected to have a surface initiated crystallization as demonstrated for the Sr glass in ref. 22 and for typical silicate bioactive glasses in refs 6 and ^8^. The surface crystallization of borosilicate glasses was also evident and will be reported shortly^7, 19^. It is therefore expected that with small particle size the risk of crystallization upon increasing temperature increases due to an increase in the number of nucleation sites with increasing the surface area to volume ratio^6, 8, 24, 25^. Glass S53B50 and P40B10 have a wider processing window than glass Sr; therefore, sintering can be achieved without the glasses enduring adverse crystallization during heat treatment.

The XRD patterns presented in Fig. 5 show the transitional behavior which led to the crystallization of the individual glasses. Figure 5(a and b) showed the onset of crystallization at temperatures between 520 °C and 540 °C while Fig. 5(c) showed crystallization transition at 480 °C. With an increase in temperature the diffraction peaks sharpen and increase in intensity related, as expected, to an increasing crystallinity of the glass. This means that glass S53B50 and P40B10 can be sintered up to 540 °C without undergoing adverse crystallization in contrast to glass Sr, which will undergo crystallization at any sintering temperatures. Furthermore it can also be seen that the density of the samples decreases as the intensity of the peaks increases with temperature. This evidently shows how crystallization effects the densification of amorphous materials.

SEM images of the cross section of the sintered samples revealed a dense microstructure which explains the high theoretical densities obtained between sintering temperatures of 480 °C–560 °C (see Fig. 6). Figure 7 shows the SEM micrographs, recorded with backscattered electrons, of the samples sintered at 600 °C for glass (a) P40B10 (b) S53B50, and (c) Sr at high magnification. There is strong evidence of crystals forming in the P40B10 glass, whereas the darker areas correspond to the crystals and the brighter areas to the remaining glass. This correlates strongly with the corresponding XRD traces, as shown in Fig. 5. SEM investigation of glass S53B50 showed almost no or minor presence of crystals. This is in agreement with the XRD pattern exhibiting low number of diffraction peaks with low intensity indicating reminiscence of a mainly amorphous matrix. The XRD patterns of glass Sr show adverse crystallization and this is clearly confirmed by the SEM micrograph of the glass which reveal high volume of crystals present on the cross section of the samples. Similar results have been reported in ref. 19 where rapid surface crystallization led to fully crystallized particles at such temperatures. In contrast to the crystallization mechanism of glass S53B50, crystals of glass P40B10 appeared to be irregularly shaped and clustered together while crystals of glass Sr exhibit a thatch like shape covering the surface of the sintered body.

One of the most important architectural feature of the scaffold is the pore size. Ideally, the pores should be at least 100 µm to enable cell migration and bone ingrowth^26–28^. In this work, scaffolds were found to have an average pore diameter in the range of 243.8 – 285.5 µm as reported in Table 4. This pore diameter range is suitable for tissue engineering application as it will not only allow good permeability of the fluids but will also promote bone ingrowth and vascularization. A second architectural feature that is of importance is the size of the interconnections. The interconnections are the pathways between the pores. Porosimetry gives indication of the size of the interconnections. Here all scaffolds have interconnections in the order of 50 µm. It was reported in ref. 29, that the minimal necessary interconnections, in bioceramics, is 20 µm, but it is most favorable for cell penetration when they are >40 µm. Interconnections over 50 µm can assure mineralized bone formation. The similarity in overall porosity obtained from calculation of the theoretical density, analysis of the µCT images and from porosimetry experiments (Tables 3 and 4) also indicates that the majority of the pores are open and interconnected which is favourable for fluid transport throughout the scaffold.

## Methods

### Glass and Powder preparation

The starting materials for the borosilicate glass designated S53B50 were analytical reagent grades of Na_2_CO_3_, H_3_BO_3_, CaCO_3_, CaHPO_4_.2H_2_O and 99.4% pure SiO_2_, while the borophosphate and phosphate glasses designated P40B10 and Sr respectively were prepared using NaPO_3_, H_3_BO_3_, SrCO_3_, CaCO_3_ and (NH_4_)_2_HPO as raw materials. Ca (PO_3_)_2_ and Sr (PO_3_)_2_ were used as precursors for the phosphate glasses and were prepared independently using a slow heating rate up to 900 °C.

The three glass compositions studied are given in Table 1. Glass S53B50 had a nominal oxide composition of 26.93SiO_2_-26.93B_2_O_3_-22.66Na_2_O-1.72P_2_O_5_-21.77CaO, while glass P40B10 and Sr had a nominal composition of (50-x) P_2_O_5_-xB_2_O_3_-20CaO-20SrO-10Na_2_O; where x = 0 (Sr) or 10 (P40B10). Proportions of relevant oxides of the starting materials were mixed together in a platinum crucible and melted at 1250 °C for glass S53B50 and 1100 °C for glass P40B10 and Sr. The melt was poured onto a graphite mould and annealed at 40 °C below their respective glass transition temperature T_g_ to release internal stresses and allowed to slowly cool down to room temperature in the annealing furnace. The obtained glass ingots were then crushed using a metallic mortar and pestle for 1 hour and sieved.

### Thermal properties

The glass transition temperature T_g_ and the onset crystallization temperature T_x_ of the glasses were determined using differential thermal analysis (SDTA, Netzch F1 JUPITER^e^) at a heating rate of 10 °C/min for particle size of 125–250 µm. The measurements were performed on 30 mg samples in platinum pans in a N_2_ atmosphere. The glass transition temperature T_g_ was taken at the inflection point of the first endotherm, obtained by taking the first derivative of the DTA curve. The crystallization temperature T_p_ was found at the maximum of the exothermic peak.

The crushed powders were charged and milled in a planetary ball mill (Fritsch Pulversette 6) using alumina balls (2 mm diameter) for 1 hour at 150 rpm, with distilled water as a grinding media. The charge to ball ratio was kept at 1:1. Dolapix C64 and PEG (polyethylene glycol) were added at 3 wt.% to the mixture as a dispersant and binder respectively. The slurries were dried in an oven at 70 °C for 24 hours. The dried powders were then sieved and a Mastersizer 2000 (Malvern Instruments, Germany) was used to measure the particle size distribution of the powders.

For the porous scaffolds, the glass powders were mixed with 60 vol.% and 70 vol.% NH_4_(HCO_3_) foaming agent. To ensure homogeneity, mixing was done in the T2F turbula mixer using 2 mm alumina balls for 30 minutes. After mixing, the resulting powders were compacted using a hydraulic press at 19.6 MPa to obtain pellets of 18.01 mm diameter and 4 mm thick.

### Sintering with/without NH_4_(HCO_3_)

Sintering of the samples was done with and without NH_4_(HCO_3_) foaming agent. The samples prepared without NH_4_(HCO_3_) were sintered using a heating rate of 10 °C/min at sintering temperatures between 480 °C and 600 °C and a holding time of 2 hours. On the other hand the samples with NH_4_(HCO_3_) were sintered for 1 hour at 540 °C for S53B50 and 500 °C for P40B10 and Sr after burning off the NH_4_(HCO_3_) foaming agent at 67 °C for 2 hours. All the samples were sintered in a Muffle furnace (Elite Thermal system BRF 18/5E-2416 + 2116) and cooled by switching off the furnace immediately after the sintering time was completed.

### Characterization of sintered samples

The density of the samples sintered without NH_4_(HCO_3_) foaming agent was determined using Archimedes principle as most of the pores are closed, whereas the density of the porous scaffolds, obtained with addition of a porogen (NH_4_ (HCO_3_)), was determined by the geometrical density (mass of the sample over its volume). The general equation used to derive the theoretical density of the sintered bodies was:

$${\rm{Theoretical}}\,{\rm{density}}\,( \% {\rm{TD}})=\frac{Calculated\,density}{Actual\,density}\,\times \,100$$

The sintered samples were further characterized using X-ray diffraction (D2 Phaser), with Cu Kα radiation. Diffractograms were collected over a 2*θ* range of 10–90°, with a step size of 0.02°. All the microstructure observations were done using scanning electron microscopy (FEI Quanta 400 FEG SEM) with attached EDS system.

Micro-computational tomography (µCT), a MicroXCT-400 (Carl Zeiss X-ray Microscopy, Inc., Pleasanton, USA) was used with tube voltage 140 kV and current 71 µA. Pixel size was 5.6 µm. Porosity analysis was done with Fiji using BoneJ plugin. µCT visualizations were done with Avizo 9.1 (FEI Visualization Sciences Group).

Mercury Intrusion Porosimetry (using an AutoPore IV 9500 V1.09, serial 833) and micro computational tomography (µCT) were performed to determine the porosity and the pore size distribution of the scaffolds. The mercury was intruded using a pressure from 0.10 to 60 000 psia at an equilibration time of 10 seconds using an evacuation pressure and time of 50 µmHg and 30 minutes, respectively.

## References

[1] Hutmacher DH (2000). Scaffolds in tissue engineering bone and cartilage. Biomaterials..

[2] Tomford WW (1995). Transmission of disease through transplantation of musculoskeletal allografts. J. Bone Joint Surg. Am..

[3] Bauer TW, Muschler GF, Reis RL (2000). Bone graft materials. An overview of the basic science. Clin. Ortho. Relat. Res..

[4] Hench LL, Splinter RJ, Allen WC, Greenlee TK (1971). Bonding mechanisms at the interface of ceramic prosthetic materials. J. Biomed. Mater. Res..

[5] Hench LL (1991). Bioceramics: From Concept to Clinic. J. Am. Ceram. Soc..

[6] Fagerlund S, Massera J, Hupa M, Hupa L (2012). T – T – T behaviour of bioactive glasses 1–98 and 13–93. J. Am. Ceram. Soci..

[7] Massera J, Fagerlund S, Hupa L, Hupa M (2012). Crystallization mechanism of the bioactive glasses, 45S5 and S53P4. J. Am. Ceram. Soc..

[8] Filho OP, Latorre GP, Hench LL (1996). Effect of crystallization on apatite-layer formation of bioactive glass 45S5. J. Biomed. Mater. Res..

[9] Fu Q, Saiz E, Rahaman MN, Tomsia AP (2011). Bioactive glass scaffolds for bone tissue engineering: state of the art and future perspectives. Mater. Sci. Eng. C..

[10] Abou Neel EA, Pickup DM, Valappil SP, Newport RJ, Knowles JC (2009). Bioactive functional materials: a perspective on phosphate-based glasses. J. Mater. Chem..

[11] Magyari K, Stefan R, Vulpoi A, Baia L (2015). Bioactivity evolution of calcium-free borophosphate glass with addition of titanium dioxide. J. Non. Cryst. Solids..

[12] Massera J, Hupa L (2014). Influence of SrO substitution for CaO on the properties of bioactive glass S53P4. J. Mater. Sci: Mater Med..

[13] Massera JJ (2015). Processing and characterization of novel borophosphate glasses and fi bers for medical applications. J. Non. Cryst. Solids..

[14] Arstila, H. Crystallization Characteristics of Bioactive Glasses. Doctoral Thesis. Åbo Akademi University (2008).

[15] Wang RM (2015). Biocompatibility and osteogenic capacity of borosilicate bioactive glass scaffolds loaded with. J. Mater. Chem. B..

[16] Locs J, Zalite V, Berzina-Cimdina L, Sokolova M (2013). Ammonium hydrogen carbonate provided viscous slurry foaming-A novel technology for the preparation of porous ceramics. J. Eur. Ceram. Soc..

[17] Kazutaka, K., Hyodo, T., Shimizu, Y. & Egashira, M. Fabrication of highly porous alumina-based ceramics with connected spaces by employing PMMA microspheres as a template. *Adv. Mater. Sci. Eng*. **2009** (2009).

[18] Brovarone CV, Verné E, Appendino P (2006). Macroporous bioactive glass-ceramic scaffolds for tissue engineering. J. Mater. Sci. Mater. Med..

[19] Massera J, Mayran M, Rocherulle J, Hupa L (2015). Crystallization behavior of phosphate glasses and its impact on the glasses’ bioactivity. J. Mater. Sci..

[20] Lowell S, Shields JE (1984). Theory of mercury porosimetry hysteresis. Powder. Tech..

[21] Qin, L., Genant, H. K., Griffith, J. F. & Leung, K. S. Advanced bioimaging technologies in assessment of quality bone and scaffold materials (eds Heilmann, U.) 289–306 (Berlin Heidelberg, 2007).

[22] Gao Z (2013). Estimating permeability using median pore throat radius obtained deom mercury intrusion porosimetry. J. Geophys. Eng..

[23] Brauer DS (2015). Bioactive Glasses — Structure and Properties. Angew. Chem. Int. Ed..

[24] Marrota BF, Buri A (1981). Nucleation in glass and differential thermal analysis. J. Mater. Sci..

[25] Ray CS, Day DE (1997). An Analysis of Nucleation-Rate Type of Curves in Glass as Determined by Differential Thermal Analysis. J. Am. Ceram. Soc..

[26] Fu Q, Saiz E, Rahaman MN, Tomsia AP (2010). Bioactive glass scaffolds for bone tissue engineering: state of the art and future perspectives. Mater. Sci. Eng. C. Mater. Bio. Appl..

[27] Karageorgiou V, Kaplan D (2005). Porosity of 3D biomaterial scaffolds and osteogenesis. Biomaterials..

[28] Hollinger OJ, Brekke J, Gruskin E, Lee D (1996). Role of bone substitutes. Clin. Ortho. Relat. Res..

[29] Wang Z (1999). Role of porous structure of the bioceramic scaffolds in bone tissue engineering. J. Mater. Sci: Mater. Med..

